# Health beliefs and (timely) use of facility-based care for under-five children: lessons from the qualitative component of Nigeria’s 2019 VASA

**DOI:** 10.1186/s12889-022-13238-1

**Published:** 2022-04-28

**Authors:** Michael Kunnuji, Robinson Daniel Wammanda, Tellson Osifo Ojogun, John Quinley, Stephen Oguche, Adeyinka Odejimi, William Weiss, Bintu Ibrahim Abba, Rebekah King, Ana Franca-Koh

**Affiliations:** 1 Department of Sociology, University of Lagos, Lagos, Nigeria; 2 Department of Paediatrics, Ahmadu Bello University/Ahmadu Bello University Teaching Hospital, Zaria, Nigeria; 3 National Population Commission, Abuja, Nigeria; 4 Social Solution International, CIRCLE Project, Rockville, USA; 5 Department of Paediatrics, Faculty of Clinical Sciences, College of Health Sciences, University of Jos/Jos University Teaching Hospital, Jos, Nigeria; 6 Department of Health Planning, Research and Statistics, Federal Ministry of Health, Abuja, Nigeria; 7 USAID Senior Monitoring & Evaluation Advisor, IPA Mobility Program/Johns Hopkins University, Baltimore, USA; 8 Social Solutions International (United States), Rockville, USA

## Abstract

**Background:**

Nigeria’s under-five health outcomes have improved over the years, but the mortality rates remain unacceptably high. The qualitative component of Nigeria’s 2019 verbal and social autopsy (VASA) showed that caregivers’ health beliefs about causes of illnesses and efficacious treatment options contribute to non-use/delay in use of facility-based healthcare for under-five children. This study explored how these health beliefs vary across zones and how they shape how caregivers seek healthcare for their under-five children.

**Methods:**

Data for this study come from the qualitative component of the 2019 Nigeria VASA, comprising 69 interviews with caregivers of under-five children who died in the five-year period preceding the 2018 Nigeria Demographic and Health Survey (NDHS); and 24 key informants and 48 focus group discussions (FGDs) in 12 states, two from each of the six geo-political zones. The transcripts were coded using predetermined themes on health beliefs from the 2019 VASA (qualitative component) using NVivo.

**Results:**

The study documented zonal variation in belief in traditional medicine, biomedicine, spiritual causation of illnesses, syncretism, and fatalism, with greater prevalence of beliefs discouraging use of facility-based healthcare in the southern zones. Driven by these beliefs and factors such as availability, affordability, and access to and perceived quality of care in health facilities, caregivers often choose one or a combination of traditional medicines, care from medicine vendors, and faith healing. Most use facility-based care as the last option when other methods fail.

**Conclusion:**

Caregivers’ health beliefs vary by zones, and these beliefs influence when and whether they will use facility-based healthcare services for their under-five children. In Nigeria’s northern zones, health beliefs are less likely to deter caregivers from using facility-based healthcare services, but they face other barriers to accessing facility-based care. Interventions seeking to reduce under-five deaths in Nigeria need to consider subnational differences in caregivers’ health beliefs and the healthcare options they choose based on those beliefs.

## Background

### Nigeria’s child health outcomes

Despite the decline in under-five mortality from 193 deaths to 132 deaths per 1000 live births between 1990 and 2018 [1], Nigeria has both the highest number of under-five deaths and under-five mortality rate (U5MR) in the world, with 858,000 deaths recorded in a single year and a U5MR of 117 deaths per 1000 live births [2]. These statistics suggest that Nigeria may have been left behind in the global progress toward the Sustainable Development Goal 3.2 of reducing U5MR to at least as low as 25 deaths per 1000 live births by 2030 [3]. A recent verbal and social autopsy (VASA) study found that major causes of under-five deaths in Nigeria include malaria, diarrhea, pneumonia, sepsis, and intrapartum injury [4]. The Nigeria 2019 VASA included a qualitative component that documented a wide range of contextual factors in health seeking for under-five children, including health beliefs—the convictions people hold about causes of illnesses in children and efficacious healthcare options [5]. This study explored major health beliefs in Nigeria and showed how these beliefs influence caregivers’ treatment options. It also documented regional variation in health beliefs and use (including timely use) of facility-based care for under-five children in Nigeria.

### What do we know from previous studies?

#### Demand- and supply-side barriers

Studies have shown that the major causes of neonatal deaths in Nigeria are severe infections, intrapartum injury/birth asphyxia, and preterm delivery [4, 6, 7], suggesting that the quality of antenatal care and delivery (including place of delivery) is key to the survival of newborns. Similarly, the quality of care given to sick under-five children affects their mortality significantly. Research has shown that use of facility-based healthcare for children increases their likelihood of surviving infancy [8]. Yet, several barriers prevent caregivers from using facility-based healthcare services during pregnancy, labor, and delivery, as well as for their sick children. Barriers to use of facilities for child healthcare may include female caregivers’ need for permission from their husbands to visit a facility and household decision-making norms which are skewed against women [5, 9]; concern about being attended to by male health workers; perception that health facilities are poorly equipped and provide poor quality care; and distance to a health facility, poor road infrastructure, and the cost of accessing care [5, 9–12]. On the other hand, higher education, being employed, living in households in the upper wealth quintiles, and having access to the media are associated with increased uptake of healthcare services for children with acute illnesses [13]. Alabi et al. [14] also found that mothers’ education, living in an urban community, and living in southern Nigeria increase the likelihood of facility delivery.

In addition, maternal and child health services in health facilities are poorly funded, poorly equipped, and inadequately staffed [15–18]. Government funding of health facilities and services is notably low, with patient out-of-pocket expenses, donor funds (from individuals and organizations), and social insurance accounting for the greater part of the expenditure on health in the country [17, 19].

Demand- and supply-side factors therefore work in conjunction to inhibit uptake of services. Poor funding results in poor quality of services in health facilities. In turn, this shapes public perceptions about the quality of services provided in health facilities and leads to low uptake of services [16].

#### Beliefs as barriers

Akogun and John (2005) documented caregivers’ beliefs in spiritual etiology of illness in children by witchcraft, a widespread belief among a minority ethnic group in northeastern Nigeria [20]. In parts of the southwest of Nigeria, another study documented widespread belief in *abiku* (children from the spirit world, who often fall ill with health conditions that do not improve with the use of biomedicine) [21]. A recent study shows that caregivers in parts of North Central (Kogi) and South East Nigeria (Ebonyi) attribute child illnesses to non-biomedical causes [9]. Hill et al. (2020) also documented a fatalistic belief among caregivers in providing care for their children, which affects acceptance of facility delivery negatively [10]. Another study found that healthcare providers’ beliefs about child healthcare often are based on myths rather than scientific evidence despite their training in biomedicine [22].

Beliefs about causes of illness inform beliefs about efficacious treatments. Caregivers choose treatments based on beliefs, and often, they use both traditional birth attendants (TBAs) and health facilities for antenatal care and delivery because they believe that combining these services gives the best treatment result (a form of syncretic belief) [23]. In providing care for sick children, caregivers often combine traditional herbal remedies and biomedicine, the first step typically being traditional herbal treatment and self-treatment [9].

### Why this study is important

Previous studies on how health beliefs shape child healthcare-seeking in Nigeria are not nationally representative and do not show regional variation. This study fills this gap by providing answers to two questions: 1. What health beliefs prevent (timely) utilization of facility-based care for under-five children in Nigeria? 2. How do these beliefs vary across regions? The answers to these questions can provide useful information about contextual specificities which can inform subnational program design and policies to address Nigeria’s unacceptably high under-five mortality. The results of the study will equip governments and organizations to move away from a “one-size-fits all” approach to program design by showing the specific health education needs of different parts of the country, with a focus on the communities with the highest numbers of under-five deaths.

### Explaining health beliefs and healthcare behavior

The health belief model (HBM) helps us understand how perceived susceptibility to a condition, perceived severity of the condition, perceived benefits of action, and perceived negative implications of action influence health-promoting action. Internal or external cues to action trigger the decision-making process to act, with perceived self-efficacy also contributing to the decision to take or refrain from a health-promoting action [24]. The HBM does not show how decision-making is affected when multiple actions are possible, which is typically the case in seeking care for children. Taking the child to a health facility for medical examination is a health-promoting behavior, but caregivers may believe that there are other health-promoting behaviors like seeking care using traditional medicine or buying drugs from a chemist/Proprietary Patent Medicine Vendor (PPMV). The ecological model also helps us understand that the beliefs people hold about efficacious solutions to perceived threats to their children’s health may be influenced by social contexts, including interactions with other members of the family, cultural factors such as local belief systems, values, traditions, and worldviews [25].

For this study, we offer an explanation that people hold different beliefs on the causes of illnesses and efficacious solutions. These beliefs determine, to a large extent, caregivers’ chosen treatment options and the course of treatment, which may involve changing from one form of treatment to another, or using a combination of different methods at the same time. In addition to individual beliefs, contextual factors shape the process of deciding on the actions to take when a child is ill. Such factors include the common health beliefs in caregivers’ communities, the health beliefs of other individuals present when care is needed, and caregivers’ knowledge of and ability to afford different treatment options.

## Methods

### Methods of data collection

This study analyzed the qualitative data from the Nigeria 2019 VASA. Kalter et al. (2011) explain that social autopsy studies, which are based on interviews aimed at documenting social, behavioral, and health systems contributors to child deaths, help program managers and policymakers identify strategies for increasing health-promoting behavior and access to and use of healthcare services [26]. While the VASA is typically quantitative, the Nigeria 2019 VASA also included a qualitative component with different categories of participants: caregivers, key informants knowledgeable about local health systems, and male and female community members.

We used data from the qualitative component of the Nigeria 2019 VASA conducted in 12 states, two from each of Nigeria’s six geo-political zones. Nigeria’s geo-political zones have six states each with the exception of the North West (with seven states and the Federal Capital Territory) and the South East (with five states). The two states selected in each zone had the highest numbers of under-five deaths during the 2018 Nigeria Demographic and Health Survey (NDHS). Within each selected state, three NDHS clusters (a cluster is typically a community) with the highest number of under-five deaths were included in the study; and in the selected clusters, two caregivers who reported the most recent deaths were interviewed if they consented to be part of the study.

In the clusters with the highest and second highest numbers of deaths in each state, two key informants—persons knowledgeable about the local health systems (typically healthcare providers and, in few cases, traditional birth attendants working and living within the communities)—were interviewed. In addition, two focus group discussions (FGDs) were conducted in each of the two clusters with the highest and second highest number of under-five deaths, with male and female community members who had lived in the community for a minimum of 12 months.

A total of 93 interviews were conducted, 69 with caregivers of under-five children who died within the five-year period preceding the Nigeria 2018 Demographic and Health Survey, and 24 with key informants. Three caregivers could not be interviewed because they had migrated and could not be located. A total of 48 FGDs were conducted, four in each of the 12 states studied.

### Research tools

The study used a semi-structured interview guide that asked participants about events preceding the death of their children, health-seeking activities, roles of family members in child healthcare, and beliefs about the cause of the death of their children, among other questions.

A semi-structured interview guide was also used to collect information from key informants on caregivers’ typical health-seeking behavior and barriers to use of health facilities. Interviews were conducted in Hausa, Igbo Yoruba, Pidgin English, and formal English, depending on interviewees’ preferences. The ages of interviewees ranged from 19 to 40 years with a mean of 29 years. The interviews had a mean duration of 33 min.

Community members who participated in FGDs discussed existing healthcare services available to under-five children in the different communities and why caregivers may/may not use facility-based care for their under-five children. Group discussions were organized for male and female community members separately. Group size ranged from six to ten, with an average of eight participants. FGDs lasted about 52 min on average.

Trained field researchers conducted all interviews and FGDs and transcribed the audio recorded interviews. Verbatim transcription of the interviews and FGDs was done. In situations where field researchers could not obtain permission to record interviews, detailed notes were taken and the interview notes were used for analysis. One member of the team conducted the analysis for this study through an iterative deductive coding, adding a few nodes as coding progressed. The analysis thus combined both deductive and inductive coding. The team used NVivo (version 12) for the coding. The lead researcher created a codebook based on major themes related to the health beliefs identified in the Nigeria 2019 VASA. The themes are spiritual causation, traditional medicine, syncretic health belief, and fatalism. The coding process produced additional themes such as “belief in biomedicine”, and “exceptions to the use of biomedicine,” “Patent Medicine Vendors and home biomedical treatment,” and “faith healing” as child nodes.

### Ethics approval

The National Health Research Ethics Committee of Nigeria’s Federal Ministry of Health and the Institutional Review Board of Social Solutions International (US) reviewed and approved the research protocol and tools. During the 2018 NDHS data collection, field researchers informed caregivers who had reported under-five deaths of the follow-up VASA study and sought their consent to participate. Only those who consented were included in the study. In addition, the research team obtained participants’ consent before interviews and FGDs were conducted, and interviews with caregivers were conducted outside hearing distance of third parties. The research team anonymized all transcripts by replacing real names with pseudonyms.

## Results

The results are presented in the order of the aggregate number of coding references in each theme, starting with the nodes with the highest to the lowest. Consequently, the themes are presented in the following order: Traditional medicine (183 references), biomedicine (119 references), spiritual causation (105 references), syncretism (63 references), and fatalism (56 references).

### Traditional medicine

#### Belief in the efficacy of traditional medicine

The study documented belief in the efficacy of traditional medicine and preference for it over other ways of seeking care for children across zones. The belief that traditional medicine is equally or more efficacious than biomedicine was documented in the South West (FGD #41–43, 45–47, 82, 84, 87, 90, 92), South South (Akwa Ibom in particular) (FGD #33, Interview #66, 70–72), the South East (FGD #29, Interview #50, 52, 56, 61, 64, 75), and the North East (FGD #15, Interview #24, 28, 30). While the study shows that caregivers in the North West also believe in the efficacy of traditional medicine (Interview #36, 37, 41), participants explained that the belief was not common and it has reduced significantly over the years (Interview #40). In the North Central zone, belief in the efficacy of traditional medicine is not widespread although it does exist (Interview #7, 16). Sometimes, this belief is illness-specific. Caregivers believe that traditional medicine is more efficacious for specific health conditions in children (FGD #29, 41) such as convulsion (FGD #29, Interview #61, 62).

In some communities in the South South and South West, people routinely administer traditional medicine prepared from roots, tree bark, and leaves (locally called *agbo*)*,* to children to produce immunity to diseases even when they show no signs of illness (Interview #75, 90). A caregiver said: “*I just gave it to the baby so that the baby can be strong.”* (Interview 75, Caregiver, Rivers).

Based on this belief in the efficacy of traditional medicine, a study participant made a case for the inclusion of traditional medicine into the country’s formal health system:

*Traditional medicine should be included in PHC [primary health care]. We cannot survive without our culture. There are concoctions in every culture that can help our children survive more. We have killed our culture with foreign culture* (FGD 45, Male, Osun).

Women frequently use TBAs for delivery in the South South (Akwa Ibom especially) and the South East, regardless of whether they have accessed antenatal care in health facilities (FGD #34–36, Interview #49, 71).

When traditional medicine fails, caregivers turn to biomedicine (FGD #7, Interview #61, 71, 92). A traditional medicine practitioner said: “*If it passes our power and must be treated in the hospital, we’ll say it is not ours. Then, we’ll send them to the hospital*” (FGD 7, Male, Plateau).

#### Traditional medicine only

The belief that certain illnesses in children can only be cured with traditional medicine is common in the South East and South West zones and in parts of the South South. The belief is less common in the northern zones. Illnesses which caregivers in Ebonyi cited as being treatable only through traditional medicine (and which would prove fatal if treated with biomedicine) include *ihe eghirigha* [multiple illnesses at once], *oke ejo onwo* [very big boil], *jadi-jadi/eriri isi/eku efor/efia* [convulsion] (FGD #25, 26, 27, 28). In Imo, participants also mentioned *nra onu, jedi-jedi, ogburo afo, nwaobro afo* [splenomegaly], and epilepsy (FGD #29, 30). In the South South, participants mentioned convulsion, *akpa, ikpakip* [stomach ulcer]*,* and *jedi-jedi* as illnesses that can be cured only with traditional medicine (FGD #36, 39). In the South West, illnesses that participants believe can be cured only with traditional medicine include *kolobo* [which turns the tongue black], measles, *eela alapaadi* [big black rashes] (FGD #45), *olo inu* [colic], and *oka ori* [sutural diastasis] (Interview #87). In the North Central, *ciwon daji* [shingles] was identified as an illness that can be cured only with traditional medicine (FGD #5).

A study participant said:

*… there is a sickness that is called ihe eghirigha. If you use an English medicine on any child suffering from it, the child will die…* (FGD 25, Female, Ebonyi).

In many communities, traditional medicine involves the use of herbs as well as spiritual powers and rituals for healing (FGD #7, 8, 16, Interview #90). If children are believed to be possessed by a demon or attacked spiritually by witches, they are treated with traditional medicine (FGD #7, 8, 10, 13, 16, Interview #16, 29). The treatment in such cases typically involves the use of incense, spiritual perfumes, and/or some rituals including the use of incisions (FGD #7, 16, Interview #90).

#### Use of traditional medicine for other reasons

The study found that many caregivers use traditional medicine not necessarily because of their belief in its efficacy, but for other reasons, the most common being its affordability and availability. Participants stated that accessing traditional medicine is cheaper in comparison with care in health facilities (FGD #1, 2, 15, 16, 26, 32, 34, 36, 37, 45, 46, Interview #29, 39, 50, 55, 56, 59, 64, 66, 71, 77, 79, 92). The traditional healers may also accept delaying payment until treatment proves effective (FGD #46), an option that health facilities do not offer. Some caregivers use traditional medicine because there are no alternatives (FGD #5, 8, 35, 41, Interview #28, 30, 40). In many communities, there are no health facilities or health facilities are too far (Interview #40, 41). This reason for using traditional medicine featured prominently in interviews and discussion in the North West and the North Central zones. A study participant in a North Central community said:

*Lack of hospitals in the village is what makes us seek traditional medicine. It’s not that we reject hospitals. No. We have none that is close to us. That is why we help ourselves with the traditional medicines. After all, since we were born, that has been the only alternative here. It is actually the lack of hospital[s] that makes us to do that. It’s not that we choose traditional over orthodox medicine* (FGD 5, Male, Plateau).

Caregivers may use traditional medicine because those around them believe it is the right choice. For instance, caregivers often feel they should take the counsel of mothers-in-law and neighbors who recommend it (FGD #29, Interview #30, 58), notably in the South East and the North East. Sometimes, the counsel comes from healthcare workers (Interview #54). At other times, other community members influence their decisions (Interview #51, 61, 66, 76). This last observation was found more in the South East and South South zones, where neighbors often tell caregivers that their children’s illnesses are only curable with traditional medicine and encourage them to seek traditional care:

*I didn’t take her to the hospital... not for lack of money but because the sickness is what people will tell you that traditional medicine will treat. People around told me it is not a hospital issue. They suggested that traditional medicine is the best and they started doing that and the child became okay and I was very happy that she was okay, it was in the morning that it [the convulsion] started again and I decided to take her to the hospital but she died on the way* (Interview 61, Caregiver, Imo)*.*

Another study participant explained:

*The other people in the house will tell you to use palm kernel oil and shea butter, [and] other things, sometimes onions. They believe that you can treat it with that and the child will be okay and it is working* (FGD 29, Male, Imo).

In other situations, caregivers use traditional medicine because there are no health workers in the facilities to attend to them (FGD #33–35). This was found in all the regions, but especially in the South South. Additional reasons cited for using traditional medicine included hostility and disrespect of healthcare providers toward caregivers (FGD #45), most commonly in the South West; and in unsafe communities in the South South, the fear of being attacked while traveling to reach a health facility, when traditional healers and TBAs are closer to them (Interview #78, 79).

Education is also a barrier for some who cannot read and may not be able to follow prescribed instructions, or they may not feel comfortable in the formal health facility setting (FGD #25, 47), as participants in the South East and South West suggested.

### Belief in biomedicine

The study found acceptance of biomedicine as an efficacious healthcare option for children across all geo-political zones. Many participants expressed confidence in its reliability in diagnosing illnesses in children through expert medical examination and laboratory tests (FGD #9, 12, Interview #91) and in the procedural administration of drugs in a measurable way (Interview #53). Study participants also said that biomedicine reduces the chances of complications that commonly result from attempts to treat children using other (nonbiomedical) methods (FGD #14, 25, Interview #75). Some participants generally consider biomedicine to be the most efficacious treatment option (FGD #20, 29, Interview #30, 34, 35), and believe that drugs provided in health facilities produce faster results than other methods of healthcare for children (FGD #14).

*In this community, we prefer to go to the hospital because that is where proper diagnosis will be carried out to know the cause of the illness. One cannot just stay at home and say he is using herbs without going for [medical] examination. That’s why we go to the hospital* (FGD 43, Male, Ekiti State).

Many caregivers who believe in the efficacy of biomedicine may not use it for their under-five children because they cannot afford it (FGD #46, Interview #2, 4, 6); there are no well-equipped health facilities in their communities (Interview #1, 28, 46), or the health facilities lack personnel or drugs (FGD #4). One caregiver explained:

*We like going to the hospital [but] there are people who like visiting* boka *[herbalist] and it is because there is no money to pay hospital bills. If there was money they would prefer to go to the hospital. The herbalist will mix plants and powers for you and sometimes they [the children] get better and many times they don’t* (Interview 2, Caregiver, Niger State).

#### Biomedical care outside health facilities

For many caregivers, a visit to a chemist/PPMV is the first-line treatment. They take their under-five children to those managing the medicine stores to “mix drugs” at the first sign of illness (FGD #26, 30, 31, Interview #51, 52, 58, 69). This practice is common in the South East but was also observed in the other zones. When drugs are mixed, the chemist sells a combination of drugs to the caregiver, depending on the amount he or she is willing to pay (Interview #61). Caregivers explained that they choose PPMVs when the illness is considered not severe (Interview #61, 70). Participants said:

*We will buy drugs from the chemist before we take the child to the hospital, especially infants. However, from the advert of the drugs, it is often said that if symptoms persist for two days we should see the doctor* (FGD 43, Male, Ekiti State).

Some take children to PPMVs on the advice of their “mother-in-law doctors,” an expression an FGD participant used to describe mothers-in-law who exercise greater power than mothers over child healthcare (FGD #32). Another reason for the choice of PPMVs is that sometimes, caregivers visit health facilities but there are no healthcare providers to attend to their children (FGD #33, 39). They therefore sometimes seek out healthcare providers at home or go to PPMVs for treatment (Interview #74–76). The practice of seeking care for children in the homes of healthcare providers is most common in the South East and South South. Another motivation for this option is the lower cost and flexible payment options of accessing care from PPMVs or at the homes of nurses (FGD #33, Interview #62, 63, 77). In a group discussion, a participant said:

*If they know the type of drugs that will cure the children, they will rather go to buy from the chemist for self-treatment, because they’ll say, the money that they would pay in the hospital will be a lot* (FGD 33, Male, Akwa Ibom State).

Study participants noted that some of the PPMVs providing medical care to children in these contexts may not have the needed training to save children’s lives, they are often not licensed to offer these services, and they sometimes sell substandard, adulterated, or expired drugs (FGD #33, 44). The study found that there is no clear demarcation between nurses and midwives providing care in their homes and PPMVs. Some have some medical training while others do not. Some registered nurses and midwives operate drug shops where they examine sick children; and other PPMVs with little or no medical education are operating drug stores where they also examine children and administer drugs and injections. Some study participants referred to them as “quacks” and expressed fear about the quality of their services (FGD #38, Interview #88, 92). Yet they may still access their medical services, even if they trust the efficacy of biomedicine, because these providers are available during night hours when health facilities are not open (Interview FGD #38, Interview #88).

The use of PPMVs and healthcare providers who practice out of their homes is most common in the South East (Imo and Ebonyi), South South (Akwa Ibom and Rivers), and South West (especially in Ekiti). It was also observed in the northern zones, but not as common as in the Southern zones.

#### Exceptions to acceptance of biomedicine

Though the study found that most people trust biomedicine, there were some caveats in some zones. Participants mentioned illnesses for which injections must not be administered, saying that injection would result in their death (FGD #5, Interview #8). Examples include *ciwon daji* [shingles] (FGD #5), diarrhea, and sunken fontanel (Interview #16). Some caregivers also consider blood transfusion a taboo and will refuse this treatment for their children (Interview #7). This belief is common in the North Central zone. In the South South and South West zones, some participants who agreed to the efficacy of biomedicine objected to immunization in children because they believed it worsens the health condition of children or might kill them (FGD #35, Interview #93). One said:

*You’ll give your child immunization and it will make you waste your money…. It makes them worse…. then you will now spend more money to buy more medication* (FGD 35, Male, Akwa Ibom).

Another participant said:

*Some people don’t believe in the uptake of the immunization, they claim it kills their children* (Interview 93, Female, Key informant, Osun State).

These objections to biomedicine are widespread in Plateau and Niger States in the North Central zone, but also were documented in Imo (South East) and Osun (South South).

### Spiritual causation

The study documented widespread belief in illnesses caused by spirit possession in all the zones. Participants believe that children may fall ill because they are possessed by some spirits (FGD #2, 13, 20, 25, 44, Interview #26, 41, 60, 69) or attacked spiritually by witches (FGD #3, 7, 8, 33, 34, 36, 42, Interview #3, 15, 16, 25, 26, 29, 37, 47, 50, 53–57, 59, 61, 66–68), in which case the preferred treatment option is traditional medicine or faith healing in a church. In Akwa Ibom where this theme featured prominently in interviews and discussions, spiritual attack is referred to as *eka satan,* which is believed to be used to charm and kill children. The belief is also common in the South East. A study participant’s words sum up how this belief influences healthcare seeking behavior:

*Yes, spiritual attack happens, because there are some babies that after one applied every form of treatment, nothing good comes from the treatment until you take the baby to the traditional medicine doctor, because laboratory equipment don’t detect that kind of sickness* (FGD 32, Female, Imo State).

Another caregiver gives insight into how the belief affects timely use of health facilities.

*In the beginning, I thought the deceased was involved in spiritual attack, and this delayed us from going to the health center* (Interview 25, Caregiver, Female, Gombe State).

Some participants believe that illness may be caused by a deity that wants the child dedicated to them (FGD #25). Some children are believed to be reincarnated. In such a situation, it is believed that the child chooses their own name spiritually, and if given other names, they become ill and may die. This is why caregivers consult spiritual healers and not health facilities (FGD #26, 32). Some believe that illness occurs because the child is an *ogbanje* child (an evil child that dies and is reborn into the same household in a cycle) (Interview #51, 52, 63). This belief was documented in the South South. In some situations, caregivers’ health-seeking decisions are guided by prophecies that they would die if they used health facilities, especially for delivery (FGD #34–36, Interview #77, 85). This was found to be common in the South South.

These beliefs explain why pregnant women choose to give birth in the church or take children to church for care or consult traditional healers rather than a health facility (FGD #25, 33, Interview #51, 52, 63). Some religious sects also discourage the use of biomedicine (FGD #30). They teach their followers about miraculous healing independent of use of medicines and immunization for children (FGD #33–35, 41, 46, 47, Interview #63, 72, 78, 85). Some register with health facilities but choose the prayer house as the preferred place of delivery because there, they can be scanned spiritually with solutions proffered to manage their spiritual problems (Interview #69, 70). Belief about a deity or *ogbanje* spirit causing fatal illness in a child is common in Ebonyi in the South East. Belief in spiritual attacks by witches is common in Ebonyi, Akwa Ibom, and Imo. The belief was also documented in the South West, Gombe, Plateau, and Jigawa but was not common in Kebbi, Bauchi, Niger, and Rivers (FGD #18, 21, 22, 23, 24, Interview #58). Where people hold these beliefs, the health facility is usually the last resort, sought only when the traditional healer’s efforts have proven futile (FGD #7). Overall, belief in spiritual causation is common in the South South, South East and South West.

### Syncretism

The beliefs caregivers, and those who may influence them, hold about efficacious treatment options are not mutually exclusive and often, individuals believe in combining treatment options. Caregivers commonly combine traditional medicine with biomedicine, often starting with the former and using the latter only as a last resort (FGD #6, 10, 14, 15, 25, 48, Interview #28, 41, 71). Conversely, caregivers may also revert to traditional medicine if they try biomedicine and find that it is not effective (FGD #14, 16, 43, Interview #26, 34, 44, 46). A female participant said: *Well, the mothers, you see, if they’ve tried the healthcare center and there is no improvement, they turn to traditional medicine”* (FGD 16, Female, Gombe). In Ebonyi, the study documented a syncretic belief that traditional medicine is useful for diagnosis while biomedicine is useful for treating the identified diseases. This explains why treatment often starts with traditional medicine and progresses to biomedicine (FGD #25, 26):

*You must use the native medicine first to ascertain what the child is suffering from* (FGD 26, Male, Ebonyi).

*What I know is that it is wrong to start the treatment of any illness with orthodox medicine because it can make the child to die. I have had an experience when I started treating a baby with orthodox medicine without knowing that orthodox medicine was not the right medicine for that particular sickness, then the child died* (Interview 50, Caregiver, Female, Ebonyi).

Showing how traditional medicine and biomedicine are combined in treating children, a caregiver said:

*You must start the treatment with traditional medicine before you know whether to use the English medicine or not…. If the child has so much sickness in the body, the traditional medicine will bring out all the sickness in the person’s body, then you will use the English medicine to treat all the sicknesses* (Interview 52, Caregiver, Female, Ebonyi).

Some caregivers also believe in a combination of spiritual rituals and biomedicine (Interview #60, 61). A caregiver held the belief that a child in need of treatment should first be taken to the church for prayers before going to a health facility (Interview #68, 69). Sometimes, caregivers use both traditional medicine and biomedicine simultaneously (FGD #10, Interview #22, 25–27, 29, 30, 41, 50, 84, 90); and for pregnant women, a common practice is to register for and attend antenatal clinic but choose to use TBAs for delivery (FGD #34, Interview #49). While simultaneous use of traditional medicine and biomedicine cuts across the geo-political zones of Nigeria, the belief that traditional medicine is most suitable for diagnosis and biomedicine is most suitable for treatment was documented in the South East zone only.

### Fatalism

The study shows that sometimes, caregivers believe that their actions cannot alter the outcome of illness in children because the fate of the child has been predetermined by forces beyond their control (FGD #34, Interview #3, 4, 29, 37, 43, 45, 52). Such children will continue to be ill until they die. A caregiver explained that she never accessed facility-based care for her sick child in the one-year period of illness because she believed that the child’s fate was determined (Interview #37). Another caregiver discontinued treatment in a health facility because she perceived that the child was meant to die. The two-year-old child had been in the hospital for 4 days when the parents decided to leave. The mother explained:

*It was already her time to die. We came back in the afternoon, and in the night around 2:00 am at night, she passed away* (Interview 17, Caregiver, Female, Bauchi).

A similar fatalistic behavior was captured in the words of a caregiver who said:

*I told them to come and remove the thing [intravenous needle], let me take her home or “is it when she dies that I will take her home?” They refused and we kept dragging [debating] until they said I should go and pay since I felt like taking her home. I went round to look for money and paid them and took her away. As we were coming back, we had not passed XX [name of town] when she died* (Interview 59, Caregiver, Female, Imo).

Another caregiver said:

*He was not getting better because it is only Allah who makes things better, even when I took him to the hospital for the malnutrition, I went three times and from then he refused taking the food supplement and from then I did not go again and accepted my fate* (Interview 37, Caregiver, Jigawa).

Fatalism is most common in the North West, North East, and South East zones. Belief in *ogbanje* also leads to fatalism in the South East. Although both belief in spiritual causation and fatalism may be connected, with the former sometimes leading to the latter, they differ because while belief in spiritual causation may lead to the use of traditional medicine, fatalism often leads to inaction or a feeble attempt at seeking care. In a typical example of how belief in spiritual causation leads to fatalism, a caregiver expressed her fatalistic views because, according to her, her son had died three times because he is *ogbanje* (Interview #52). Another mother’s fatalism was reflected in her belief that her child was charmed (Interview #68). Experience of child death reinforces fatalism in caregivers when they feel they have done everything they could possibly do to prevent the death (Interview #10, 32, 58) as evidence shows in the North Central, North East and South East.

## Discussion

**Fig. 1:**
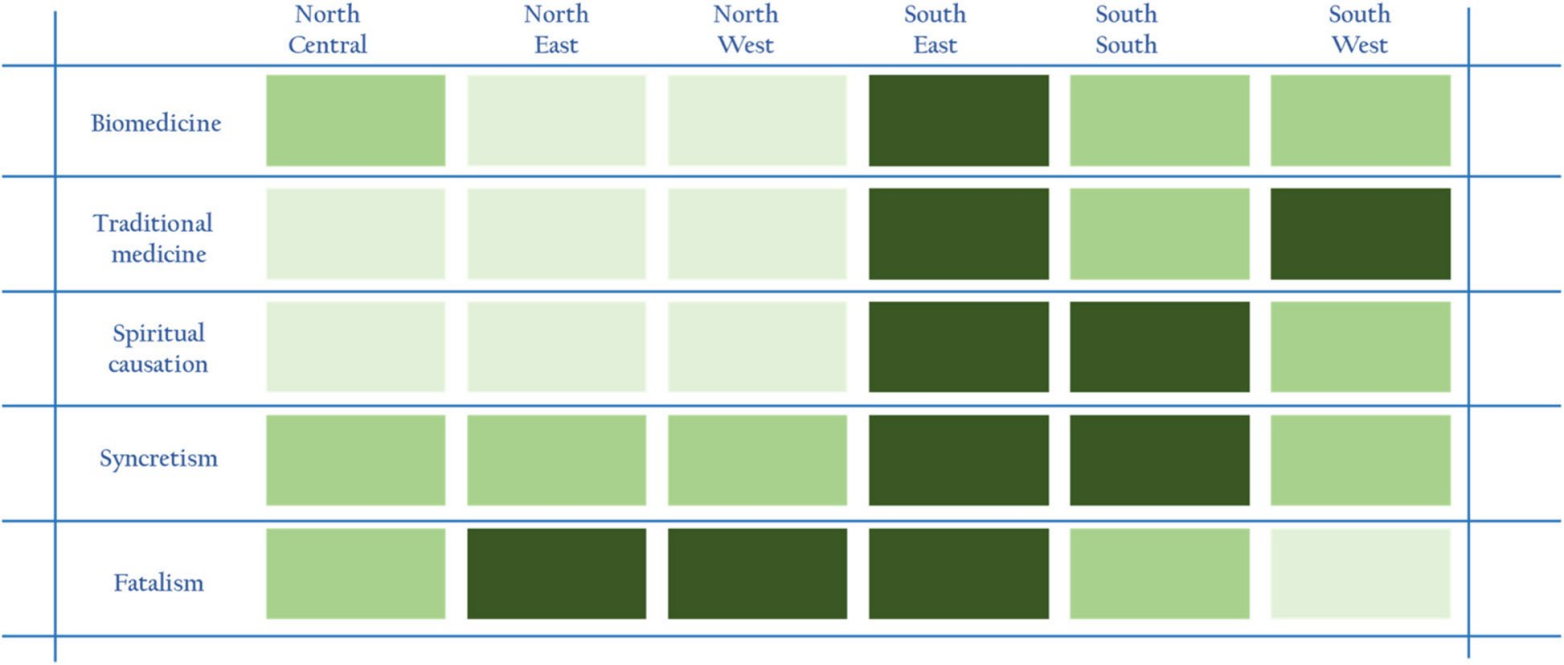
Chart showing regional spread of health beliefs in Nigeria. Note: Darker shades suggest prevalence of beliefs that do not promote use of facility-based care

Figure 1 below presents a summary of the different beliefs across the six geo-political zones and their relative impact on health-seeking behavior. Three shades of green were used to represent the prevalence of beliefs that do not promote use of facility-based care. The darkest shade under a particular theme implies that all sources of data in the study support widespread beliefs that discourage the use of facility-based healthcare. The lighter shade of green represents the existence of beliefs on a given theme but not supported by all three sources of data, suggesting that they may not be as widespread as observed in zones with the darkest shade of green. The lightest shade of green represents the least evidence found of beliefs that discourage use of facility-based care.

As Fig. 1 shows, based on health beliefs, the South East shows very strong tendency against seeking facility-based care, followed by the South South and the South West. This order is consistent with the ordering of southern zones by under-five mortality, with 75, 73 and 62 deaths per 1000 live births in the South East, South South and South West, respectively [1]. On the other hand, the general health beliefs in the North Central largely zone support seeking facility-based care, The North East and North West zones, where fatalism has stronger influence, have the highest under-five mortality rates of 187 and 184 deaths per 1000 live births respectively [1]. We note, however, that apart from beliefs, other factors like availability, accessibility, affordability and perceived quality of care are other factors that shape caregivers’ healthcare choices for their under-five children.

### Beliefs by zone

Evidence from this study shows that health beliefs vary significantly across zones. While belief in the efficacy of biomedicine is widespread across all zones in Nigeria, caregivers often do not seek biomedical care at health facilities, turning instead to PPMVs and trained healthcare workers who provide services in their homes in the southern zones where this is most common, sometimes because of affordability and ease of access. The study also found objections to the use of biomedicine for various reasons in the North Central, South East and South West zones.

The study documented widespread belief in the efficacy of traditional medicine in the South East and South West zones. In the South South (Akwa Ibom in particular), women clearly preferred TBAs and churches for delivery, indicating a strong belief in the spirituality of childbirth.

Syncretic health beliefs are more widely accepted in the South East, (especially Ebonyi State). Fatalism is common in the North West, North East, South East and parts of the South South. A previous study documented the existence of fatalism [10], but this study offers more detail in how this varies across zones.

The study also shows that health-seeking behaviors may be affected by factors other than just health beliefs. For instance, in the North East, despite a general preference for biomedicine, caregivers often start with herbal medicine, proceed to PPMVs, and finally go to health facilities because of the high cost of care associated with facility-based care. As observed by Edeme et al. (2014), poor household income is associated with higher rates of neonatal death [27]. Communities with high levels of poverty may evolve beliefs around the efficacy of alternative medicines because of reduced access to facility-based care.

**Fig. 2:**
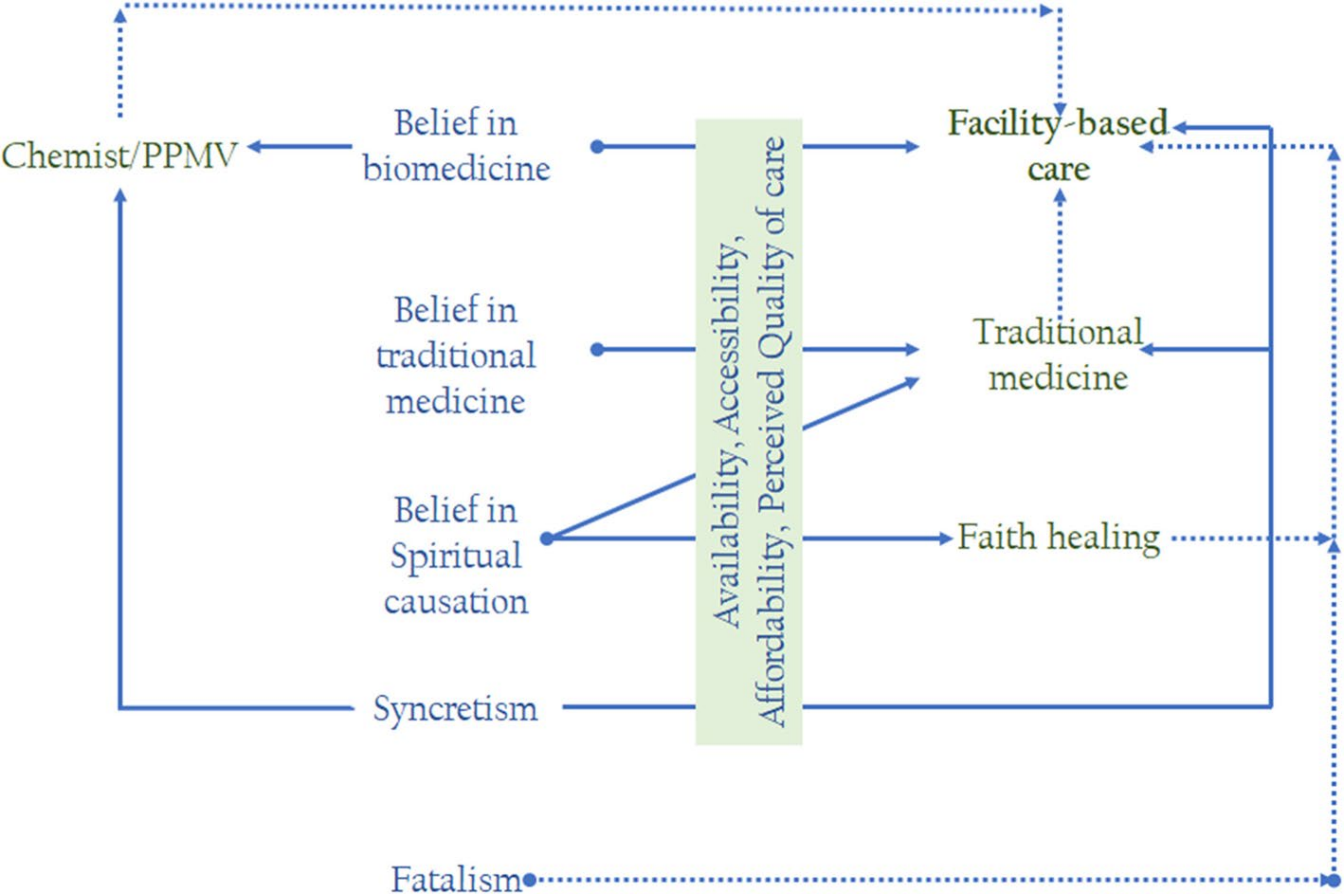
Health beliefs and health-seeking behavior. Note: Solid lines represent typical choices, while dotted lines represent probable choices/actions

In Akwa Ibom, reasons given for the preference for TBAs include the negative attitude of healthcare providers or their non-availability at night, in addition to the cost of accessing care. We argue that communities build trust in available and affordable alternatives to the ideal. An observation deserving attention is that PPMVs and trained health workers provide some services in their shops and homes. This shows that there are existing opportunities for the incorporation of informal service provision into Nigeria’s maternal and child healthcare system in order to achieve greater reach. While PPMVs operate in most communities in Nigeria, home service by trained healthcare providers is more prevalent in the South South and South East. Many caregivers prefer this arrangement because it is cheaper and offers flexible payment options, in addition to being closer and available at night when many facilities are closed to patients. Fig. 2 provides a post-results diagrammatic framework for understanding how caregivers’ beliefs [and those of close associates likes mothers-in-law and neighbors] shape their health-seeking behavior when their under-five children are ill, similar to the explanation provided in the ecological model on the roles of family members, cultural beliefs and worldviews [25].

Caregivers typically only seek out health facilities for severe illnesses, even if they believe in biomedicine, as Fig. 2 shows. The chemist/PPMV is a likely choice, though children may still end up in health facilities if their health conditions do not improve after treatment. Caregivers who adopt faith healing and traditional medicine as the first treatment options for their children may also resort to facility-based healthcare if no improvement is observed. This often means that children are not taken to a facility until their illness has progressed to a point where effective treatment is challenging or no longer viable, leading community members to believe that facility-based healthcare is less effective than traditional medicine at home and other alternatives. The limited capacity of health facilities to address severe cases in many communities further complicates the outcomes of cases taken to such facilities. This shows the need for strengthening maternal and child health policies and public health programs to address this cycle of delayed uptake of services when child illnesses are easier to manage, and poor treatment outcomes when severe cases are presented to health facilities with limited capacity for handling severe cases. Health facilities need to be better positioned to save children’s lives. This will require greater attention to the quality of healthcare provided at all levels of care, including the PHC level.

The findings agree with the HBM on the role of perceived severity of an illness in the decision to adopt health-promoting behaviour [24]. The present study however, shows how beliefs about causes of illnesses and efficacious treatments vary, resulting in interpreting different choices as health-promoting.

The study provides evidence that many who believe in the efficacy of biomedicine may access healthcare from PPMVs instead of formal health services. The requirements for a PPMV to obtain a license from the Pharmacy Council of Nigeria includes the following: literacy in the English language; being 21 years or older; and being of good character based on the attestation of two satisfactory referees. Having a pharmacy technician’s certificate is an advantage, but not required. Given these fairly loose requirements, PPMVs in Nigeria have a range of qualifications, including persons with some medical education (but not pharmacists), persons with a pharmacy technician’s certificate, and some who have neither a medical nor pharmacy technician’s certificate [28]. A significant proportion of PPMVs apprenticed under more experienced PPMVs [29, 30], and they likely have a broad range of capacities that can be built upon. With training, based on their roles as stipulated in the Nigeria Task Shifting and Task Sharing Policy, PPMVs will have enhanced capacity to conduct their usual tasks of examining and diagnosing children and prescribing medications, contributing to better outcomes for children in their care. An early priority in strengthening PPMV capacity would be to document their training needs to effectively identify and treat the common killers of under-five children which are malaria, diarrhea, pneumonia, and sepsis.

## Limitations of the study

The study is qualitative and the findings cannot be generalized to a wider context beyond the study locations, although insights may be gained from the lessons learned from the findings. Other cultural factors such as patriarchy and gender roles in child care, access to quality healthcare and supply-side barriers to use of child care services may be implicated in timely use of facility-based care for under-five children. The factors explored in this study may also have varying effects on timely use of facility-based care, an issue this study was not designed to address.

## Conclusion

Health beliefs influence caregivers’ healthcare choices during pregnancy and when under-five children are sick. These beliefs vary across zones in Nigeria. Therefore, addressing Nigeria’s high under-five mortality burden requires policies and interventions that are tailored to each zone and potentially to each state. Generally, VASAs are helpful for developing and revising policies and the design of appropriate programs. Nigeria’s 2019 VASA, and the qualitative component in particular, offers lessons beyond the biomedical to better understand the social causes of under-five deaths. It shows how different contexts shape child healthcare across the country. Nigeria will benefit from addressing health beliefs as barriers to facility-based healthcare through specific zone- (or state-) level health education programs that target community leaders, community members, and healthcare providers at all levels. Health financing or funding for child survival can also be channeled into high-level advocacy for policymakers to commit to implementing zonal- and state-level health programs. As Okonofua et al. (2011) argued, advocacy has been proven effective in getting lawmakers to commit to free, comprehensive maternal and child health services [31]. However, as this study has shown, out-of-pocket health expenses continue to be a barrier to use of facility -based healthcare across the zones, even among those who believe in the efficacy of biomedicine [32].

We also argue that community-level healthcare needs to be strengthened across Nigeria, although zonally adapted. In line with Uneke et al.’s (2014) argument in favor of community-based participatory interventions to strengthen free maternal and child health programs in Nigeria, we believe that strengthening community-level healthcare will require efforts from governments and community stakeholders [32]. Communities can be involved in addressing local beliefs and practices in ways that better promote the health of under-five children. Community stakeholders can also be organized into watch groups that will demand high-quality, respectful maternal and child care from healthcare providers/health facilities. Religious leaders also need to be involved in connecting health beliefs with religious beliefs in ways that promote safe deliveries and healthy children.

Nigeria lags behind the rest of the world in the bid to ensure healthy lives and promote wellbeing for all, and for under-five children in particular. Catching up requires doubling current efforts in terms of investment in subnational level healthcare programs that will significantly impact local caregivers’ healthcare-seeking behavior for their under-five children.

## Data Availability

The data that support the findings of this study are available from the National Population Commission, Abuja, Nigeria but restrictions apply to the availability of these data, which were used under license for the current study, and so are not publicly available. Data are however available from the authors upon reasonable request and with permission of the National Population Commission.
